# The role of age, sex, anthropometry, and body composition as determinants of physical fitness in nonobese children aged 6–12

**DOI:** 10.7717/peerj.8657

**Published:** 2020-03-17

**Authors:** Chiara Milanese, Marco Sandri, Valentina Cavedon, Carlo Zancanaro

**Affiliations:** Laboratory of Anthropometry and Body Composition, Department of Neurosciences, Biomedicine and Movement Sciences, University of Verona, Verona, Italy

**Keywords:** Standing broad jump test, 30-m dash test, Flamingo balance test, Seated chest pass test, Random forests

## Abstract

**Purpose:**

The determinants of physical fitness in children have been given limited attention. In particular, the relative role of chronological age, sex, anthropometry, and body composition in physical fitness of children has been barely investigated. This cross-sectional study investigated determinants of physical fitness using a set of predictive variables including, in addition to chronological age and sex, a large panel of anthropometric measurements as well as body composition. The study was carried out in a convenience sample of children aged 6–12 participating in a summer camp.

**Methods:**

One-hundred-ninety-three children (128 males) fulfilled all requirements and entered analysis. Health-related physical fitness components (speed, muscular power and balance) were explored by means of field tests, namely the 30-m dash test for running speed, the standing long jump and the seated chest pass test for lower limbs and upper body muscular power, respectively, and the flamingo balance test for static balance. Determinants of physical fitness were investigated by regression analysis using chronological age, sex, anthropometry, and body composition in a hierarchical approach. To minimize the expected effect of collinearity in predictor variables, an original statistical approach using Random Forests analysis was adopted.

**Results:**

Age predicted 45.2%, 43.6%, 35.6% and 25.6%; and sex 9.5%, 10.7%, 6.3% and 2.0% of variance in the 30-m dash, seated chest pass, standing long jump, and flamingo balance test, respectively. Anthropometry and body composition explained a limited or no percentage of variance. The adjusted *R*^2^ (root mean square error) was 0.61 (0.31 s), 0.45 (0.32 m), 0.58 (0.15 m) and 0.41 (0.75 logs) for the 30-m dash, seated chest pass, standing long jump, and flamingo balance test, respectively making these models useful when physical fitness tests are not feasible.

**Conclusions:**

We highlighted the respective role of chronological age, sex, anthropometry, and body composition in physical fitness of children in the wide age range 6–12 years. Data confirm and expand on previous literature by showing with a strictly conservative statistical approach that chronological age is a main determinant of physical fitness of both boys and girls, sex playing a limited role. The role of anthropometry was even less important, and no role was found for body composition. These findings should be considered when planning/implementing motor development or physical education programs.

## Introduction

Physical fitness can be defined as the capacity to perform physical activity (Coppens et al., 2019). While physical activity refers to any movement produced by muscle contraction, physical fitness involves a full range of physiological and psychological qualities (Zaqout et al., 2016) representing a set of attributes that people have or achieve (Caspersen, Powell & Christenson, 1985). Physical fitness includes performance-related fitness (which refers to those components of fitness that are necessary for optimal work or sport performance (Shephard & Bouchard, 1994; Artero et al., 2010)) and health-related fitness (which considers the ability to perform daily activities). Given the increasing prevalence of overweight/obesity and the reduction of habitual physical activity early in age (World Health Organization, 2000), the investigation of prepubertal children in respect of physical fitness and anthropometry and body composition is especially important for effective intervention such as reduced energy intake, increased physical activity and decreased sedentary behavior (Martin et al., 2018). In fact, it has been found that children who were fit and active at 10 years of age have had better physical fitness at 6 years of age vs. peers who were less fit and sedentary (De Souza et al., 2014). Also, the level of physical fitness has been positively associated with cognitive function and academic achievement in children (Donnelly et al., 2016) as well as higher risk of developing cardiovascular diseases, mental disorders, and skeletal problem later in life (Ruiz et al., 2009). Finally, physical fitness is of interest to pediatric exercise science insofar it is involved in sports and games practiced by the pediatric population (Carter & Micheli, 2011). Recognized components of health-related fitness include cardiorespiratory fitness (e.g., submaximal exercise capacity, maximal aerobic power etc.), musculoskeletal fitness (e.g., muscular power, strength, endurance etc.), and motor fitness (e.g., balance, speed of movement etc.) (Ruiz et al., 2009; Ortega et al., 2015).

The determinants of physical activity in children have been investigated to some extent (Sallis, Prochaska & Taylor, 2000; Van Der Horst et al., 2007; Craggs et al., 2011); however, data on determinants of physical fitness components are limited (Pissanos, Moore & Reeve, 1983; Butterfield, Lehnhard & Coladarci, 2002; Burns et al., 2015). Due to either limited number or narrow age range of participants results presented in the quoted papers were inconclusive, thereby limiting their impact. More recently, a large study was conducted on the determinants of physical fitness in European children (Zaqout et al., 2016) showing the largest effect size (partial eta squared) for age and sex. However, this study did not consider two potentially relevant determinants namely, body composition (which has been shown to be associated with physical fitness (Moliner-Urdiales et al., 2011; Smith et al., 2014)) and anthropometry (apart from body mass index), which has some relationship with fundamental movement skills in early infancy (Butcher & Eaton, 1989) and performance in motor tasks (Malina, Bouchard & Bar-Or, 2004). Identifying determinants of physical fitness in prepubescent boys and girls would improve professionals planning and implementation of motor development or physical education programs for children.

Based on the above, the aim of this work was to assess, by means of regression analysis, the respective role of chronological age, sex, anthropometry, and body composition in the development of physical fitness in a large sample of children aged 6–12. The weight status has been associated with decreased motor proficiency in childhood (Okely, Booth & Chey, 2004; Lubans et al., 2010; Lopes et al., 2012; Marmeleira et al., 2017) and excess body fat may be a confounding factor when evaluating performance in physical fitness tests in children (Institute of Medicine (IOM), 2012). Accordingly, nonobese children were only included in the study.

Physical fitness can be objectively and accurately measured through sophisticated laboratory-based test equipment. Unfortunately, this is often not feasible due to the high cost, necessity for qualified technicians and time constraints. Thus, generally, physical fitness is assessed in children and adolescents using physical fitness field tests. These tests are relatively easy to administer for the trained operator, involve limited equipment and personnel, and demonstrate good validity and reliability (Ortega et al., 2015).

Four tests were selected to assess physical fitness, exploring basic components of physical condition and motor development namely, the 30-m dash test for running speed, the standing long jump and the seated chest pass test for lower limb and upper body muscular power and the flamingo balance test for assessing static balance. The selection of the tests was aligned with available literature on children combining musculoskeletal component (i.e., power/strength) and motor component (balance and speed movement) (Ruiz et al., 2009). The tests battery focused on common activities such as running, jumping and throwing that are included in most children’s everyday play activities. Furthermore, test selection was based on practical considerations regarding age-appropriateness and, user-friendliness among children aged 6–12, available time for testing, and compatibility with ambient conditions. This led to some limitation in the number and type of tests performed by children. In particular, we did not test cardiorespiratory fitness, which is a limitation of the work.

Beyond yielding information on the determinant(s) of the dependent variable, regression analysis produces predictive equations able to estimate the dependent variable in subjects which were not included in the original sample. Such an estimate is amenable to comparison with measured values of the same variable in individuals, thereby allowing inference on the individual’s position vs. the reference population.

In this work, the following hypotheses were tested in a sequential approach: (1) physical fitness improves with chronological age; (2) sex affects physical fitness components independently of chronological age; (3) anthropometry or body composition contributes to physical fitness independently of age and sex. When testing hypothesis (3) the presence of a high degree of correlation between potentially predictive variables (i.e., multicollinearity) is expected, which may flaw the resulting standard regression model. This issue has been generally underscored in previous works. To minimize the effect on predictive models of multicollinearity in the data, the Random Forests (RFs) analysis (Breiman, 2001) was used in this work for variable selection using the method of minimal-depth variable importance estimation (Ishwaran et al., 2010). Variable importance measures yielded by RFs cover the impact of each predictor variable individually as well as in multivariate interactions with other predictor variables. The method of minimal-depth is able to estimate a variable importance threshold showing the ability to correctly identify predictive covariates.

## Materials and Methods

### Participants

The study protocol was in accordance with the declaration of Helsinki and received approval from the Institutional Review Board of the University of Verona (Prot. N. 290/2012). A convenience sample of 272 nonobese children attending the summer camp organized by the Faculty of Motor Sciences of the University of Verona over four consecutive years participated in the study. All children and their parents were thoroughly informed about the purposes and contents of the study, and written informed consent was obtained from at least one parent. To improve inclusion, all children participating in the summer camp were admitted to the measurement procedures. For inclusion in the analysis group the following criteria were adopted. Inclusion criteria: age 6–12 years and parental consent; exclusion criteria: inability to effectively perform fitness tests and, for the reasons stated in the Introduction, obesity. To define obesity and overweight, the BMI cutoffs proposed in Cacciari et al. (2006) for children of northern-central Italy were used. Seventy-nine children were excluded from analysis because of lack of complete anthropometric measurements. Accordingly, data from 193 children (128 males, M and 65 females, F) were used for the analysis. In the whole sample, overweight subjects were 19 (9.8%). Compared to the reference population (Cacciari et al., 2006), children in the sample showed the following percentages falling above the 75th (95th) percentile for body mass, stature and BMI, respectively: 22.8 (1.0), 28.0 (6.2) and 16.6 (1.5). No participant has had menarche or pubarche at the time of measurement. Age distribution (in tertiles) was as follows: 6–7 years, *n* = 60 (M, 42 and F, 18); 8–9 years, *n* = 64 (M, 38 and F, 26); 10–12 years, *n* = 69 (M, 48 and F, 21). Measurement sessions were taken in June/July. When a subject underwent more than one measurement session (e.g., on two or more years of summer camp participation), the first eligible one (i.e., that taken on the first year of summer camp participation) was considered in analysis.

### Anthropometry and body composition

Prior to physical fitness testing, body mass was assessed in light clothing and without shoes to the nearest 0.1 kg using a certified electronic scale (Tanita electronic scale BWB-800 MA; Wunder SA.BI. Srl, Milan, Italy). Stature was measured to the nearest 0.01 m using a Harpenden portable stadiometer (Holtain Ltd., Crymych, Pembs, UK). The body mass index (BMI) was calculated as kg/m^2^. Linear anthropometry measurements were taken on the right side of the body according to Lohman, Roche & Martorell (1988). Skinfold thickness was taken at five sites (triceps, subscapular, thorax, abdominal, front thigh) with a Harpenden caliper (Gima, Modena, Italy) according to standard procedures (Norton & Olds, 1996). Measurements were taken in duplicate, and the average of the two readings was the measure. If the two measurements differed by more than two mm, a third measurement was taken, and the two closest were then averaged and recorded as the score. All anthropometry measurements were taken by the same expert operator to ensure consistency. The equation proposed by Dezenberg and colleagues (Dezenberg et al., 1999) using triceps skinfold as the predictor, was used to estimate body fat mass (FM) and fat-free mass (FFM); this equation has been validated against dual-energy X-ray absorptiometry, showing a coefficient of determination (*R*^2^) = 0.94 (SEE, 0.34 kg).

### Physical fitness tests

The measuring took place during the summer camp physical activities between 9 am and 12 am. All measurements were taken by the same expert operator to ensure consistency. The children were tested individually, and each test item was explained and demonstrated before the child started. Participants were given verbal encouragement and support throughout the testing procedure. The sequence of tests was the same for all participants that is, flamingo balance, standing long jump, seated chest pass and 30-m dash. Before testing, all participants conducted a 10 min standardized warm-up, which included light running followed by different activities (side steps, back-wards run and stretching).

The flamingo balance test, testing static balance was performed as follows: the subject, with eyes closed, had to stand on one leg (preferred leg) on a wooden block lying longitudinally of the set dimensions (30 × 20 × 10) and the other leg was bent backwards. The test’s score is the time passed from the start to the moment the bent leg touches the floor for the first time Fjørtoft (2000); maximal duration of the test was set to 1 min.

For the standing long jump test, testing lower limbs muscular power (Adam et al., 1988), the child stands behind a line marked on the ground with feet slightly apart. Upon a signal, the child swings the arms backward and forward and jumps with both feet simultaneously as far as possible. The test item score is the distance between the starting line and the landing position. The seated chest pass test, testing upper body power (Mayhew et al., 1997), consists of pushing a medicine ball (1 kg from 6 to 7 years of age and 2 kg from 8 to 12). In the present study we chose 1 kg for children aged from 6 to 7 years (Davis et al., 2008) and 2 kg for children aged 8–12 (Loko et al., 2000; Luz et al., 2018). The subject assumes a seated position on the floor with their head, shoulder and lower back against the wall; the subject throws the ball as far as possible without the head, shoulder and hip moving from the wall. A tape measure is used to measure the distance from the wall to where the ball landed. The 30-m dash test, testing running speed (Loko et al., 2000), was assessed in a stationary starting position; children started on a vocal command and accelerated at maximum effort. The outcome is the time of the subject’s running the 30-m distance; time is fixed with a commercial gstopwatch to the nearest 0.1 s.

For each test three trials were performed (with adequate rest between trials), the score being the average of the three trials and the best performance. The average performance was used as the primary outcome in analysis because it was deemed to be more representative of the actual fitness level of children in their ecological contest. The best performance was considered as well, as this is a frequently used outcome in the literature.

### Statistical analysis

The Shapiro–Wilks W test was used to assess the normality of data distribution. This analysis suggested applying log-transformation to the performance of the flamingo balance test; accordingly, log-transformed data for this variable were used in all analysis. Statistical distribution of numerical variables was summarized as mean ± SD. The two-sample *t*-test was used to test the hypothesis about the absence of a statistically significant difference between the means in two unrelated groups. The pairwise association between physical fitness test scores, age, and anthropometric and body composition variables was assessed using the Pearson’s correlation coefficient, *r*.

To investigate predictors of performance in physical fitness tests, the effect of age was estimated using a linear regression model ${M_1}$ and then removed from data calculating the residuals of ${M_1}$. Age in months was used in ${M_1}$ in order to better exploit age variability in the sample. In the second step, the effect of sex was estimated and removed from data fitting a regression model ${M_2}$ on the residuals of ${M_1}$. In the last step, using the age- and sex-free residuals of model ${M_2}$, anthropometric predictors of performance outcome were identified with RFs (Ishwaran et al., 2010) and a subset of predictors was selected applying a thresholding rule. The predictive power of covariate(s) was also estimated using repeated cross-validation. At the end of replications, the average value of the coefficient of determination, *R*-squared (${R^2}$), was calculated. Negative or null values of ${R^2}$ indicate a lack of predictive power. The predictive equations for performance in the four physical fitness tests were estimated using linear regression models with age, sex and the selected anthropometric variables as explanatory variables and the overall predictive power was evaluated in-sample by adjusted ${R^2}$ and root MSE. According to Cohen (1992) the *R*^2^ effect size in multiple regression was interpreted as 0.02 small, 0.13 medium and 0.26 large. All statistical analyses were performed using R v. 3.3.1 (R Core Team, 2014) with the Random Forest SRC package v. 2.3.0. A *P*-value equal or less than 0.05 was considered statistically significant.

## Results

The mean values for the investigated demographic, anthropometric, and body composition variables in boys and girls, and in the study group as a whole are summarized in Table 1. All individual skinfolds were significantly greater in girls (triceps, subscapular and abdominal skinfold: *P* < 0.05; thorax and front thigh skinfold: *P* < 0.001) as well as the sum of five skinfolds (*P* < 0.001). Percentage FM was higher in girls (*P* < 0.01). Body circumferences were similar in boys and girls as well as lengths and widths with the exception of the wrist, knee and ankle breadth, which were larger in boys (*P* < 0.05, *P* < 0.01 and *P* < 0.001, respectively).

**Table 1:** Descriptive statistics for demographic, anthropometric, and body composition variables of the participant boys and girls, and the whole study group (mean ± SD).

Variable	Boys (*n* = 128)	Girls (*n* = 65)	Aggregate (*n* = 193)
Demographic			
Age (mo)	104.6 ± 22.1	104.0 ± 22.3	104.4 ± 22.1
Stature (cm)	134.4 ± 11.6	133.5 ± 12.8	134.1 ± 12.0
Body mass (kg)	31.6 ± 8.6	32.0 ± 9.6	31.7 ± 8.9
BMI (kg/m^2^)	17.1 ± 2.3	17.6 ± 2.8	17.3 ± 2.5
Skinfold (mm)			
Triceps	11.4 ± 3.8	14.2 ± 4.6***	12.3 ± 4.3
Subscapular	6.6 ± 2.7	8.1 ± 3.8**	7.1 ± 3.2
Thorax	8.1 ± 4.2	9.4 ± 4.4*	8.5 ± 4.3
Abdominal	10.2 ± 6.3	12.4 ± 6.5*	10.9 ± 6.5
Front thigh	15.9 ± 5.8	21.2 ± 8.1***	17.7 ± 7.1
Sum of skinfolds	52.2 ± 21.0	65.3 ± 25.8***	56.6 ± 23.5
Circumference (cm)			
Arm (relaxed)	20.3 ± 2.5	20.6 ± 2.6	20.4 ± 2.6
Wrist	14.0 ± 1.2	13.7 ± 1.1	13.9 ± 1.2
Waist	60.0 ± 6.4	58.9 ± 6.6	59.6 ± 6.5
Hip	71.0 ± 8.1	73.0 ± 8.9	71.7 ± 8.4
Length (cm)			
Acromion-elbow	27.8 ± 3.1	27.7 ± 3.1	27.8 ± 3.1
Radiale-stylion	21.2 ± 2.3	20.9 ± 2.4	21.1 ± 2.3
Height (cm)			
Throcanterion to floor	30.1 ± 3.7	30.3 ± 3.9	30.2 ± 3.8
Tibialelaterale to floor	36.9 ± 4.1	36.8 ± 4.2	36.9 ± 4.1
Breadth & Depth (cm)			
Transverse chest	20.1 ± 2.0	20.0 ± 2.4	20.1 ± 2.2
Anterior–posterior chest	14.5 ± 1.4	14.2 ± 1.7	14.4 ± 1.5
Elbow	5.3 ± 0.7	5.2 ± 0.6	5.3 ± 0.7
Wrist	4.4 ± 0.5	4.3 ± 0.4	4.4 ± 0.4
Knee	8.2 ± 0.7	7.9 ± 0.8**	8.1 ± 0.8
Ankle	6.0 ± 0.6	5.7 ± 0.5***	5.9 ± 0.6
Body composition			
Fat mass (kg)	7.5 ± 3.7	8.5 ± 4.2	7.8 ± 3.9
Percent fat mass (%)	22.6 ± 5.5	25.3 ± 5.4**	23.5 ± 5.6
Fat-free mass (kg)	24.0 ± 5.0	23.5 ± 3.7	23.8 ± 5.3
Percent fat-free mass (%)	77.4 ± 5.5	74.7 ± 5.4**	76.5 ± 5.6

**Notes:**

* 0.01 < *P* ≤ 0.05.

** 0.001 < *P* ≤ 0.01.

*** *P* ≤ 0.001 vs. boys.

The mean values for the best and average performance of boys and girls in physical fitness tests, and in the study group as a whole are presented in Table 2. Boys performed significantly better than girls in the standing long jump test (1.37 vs. 1.26 m and *P* = 0.002), the seated chest pass test (2.44 vs. 2.16 m and *P* < 0.001) and the 30-m dash test (4.72 vs. 4.46 m/s and *P* < 0.001). Girls showed better mean scores in the flamingo balance test than males, but the difference was borderline statistically significant (*P* > 0.05). Overweight children did not perform significantly different from normal weight ones; accordingly, further analysis was performed in the whole sample of 193 children.

**Table 2:** Best (B) and average (A) performance in fitness tests across three trials in boys and girls aged 6–12 and in the whole study group (mean ± SD).

Physical fitness test		Boys (*n* = 128)	Girls (*n* = 65)	Aggregate (*n* = 193)
30-m dash (m/s)	B	4.85 ± 0.50	4.59 ± 0.43***	4.76 ± 0.49
	A	4.72 ± 0.52	4.46 ± 0.42***	4.63 ± 0.50
Standing long jump (m)	B	1.43 ± 0.22	1.31 ± 0.22***	1.39 ± 0.23
	A	1.37 ± 0.23	1.26 ± 0.23**	1.33 ± 0.23
Flamingo balance (s)	B	15.52 ± 15.02	20.27 ± 17.84	17.12 ± 16.14
	A	11.32 ± 12.56	14.88 ± 14.21	12.52 ± 13.21
Seated chest pass (m)	B	2.58 ± 0.56	2.29 ± 0.51***	2.48 ± 0.56
	A	2.44 ± 0.55	2.16 ± 0.49***	2.34 ± 0.55

**Notes:**

** 0.001 < *P* ≤ 0.01.

*** *P* ≤ 0.001 vs. boys.

**Figure 1:**
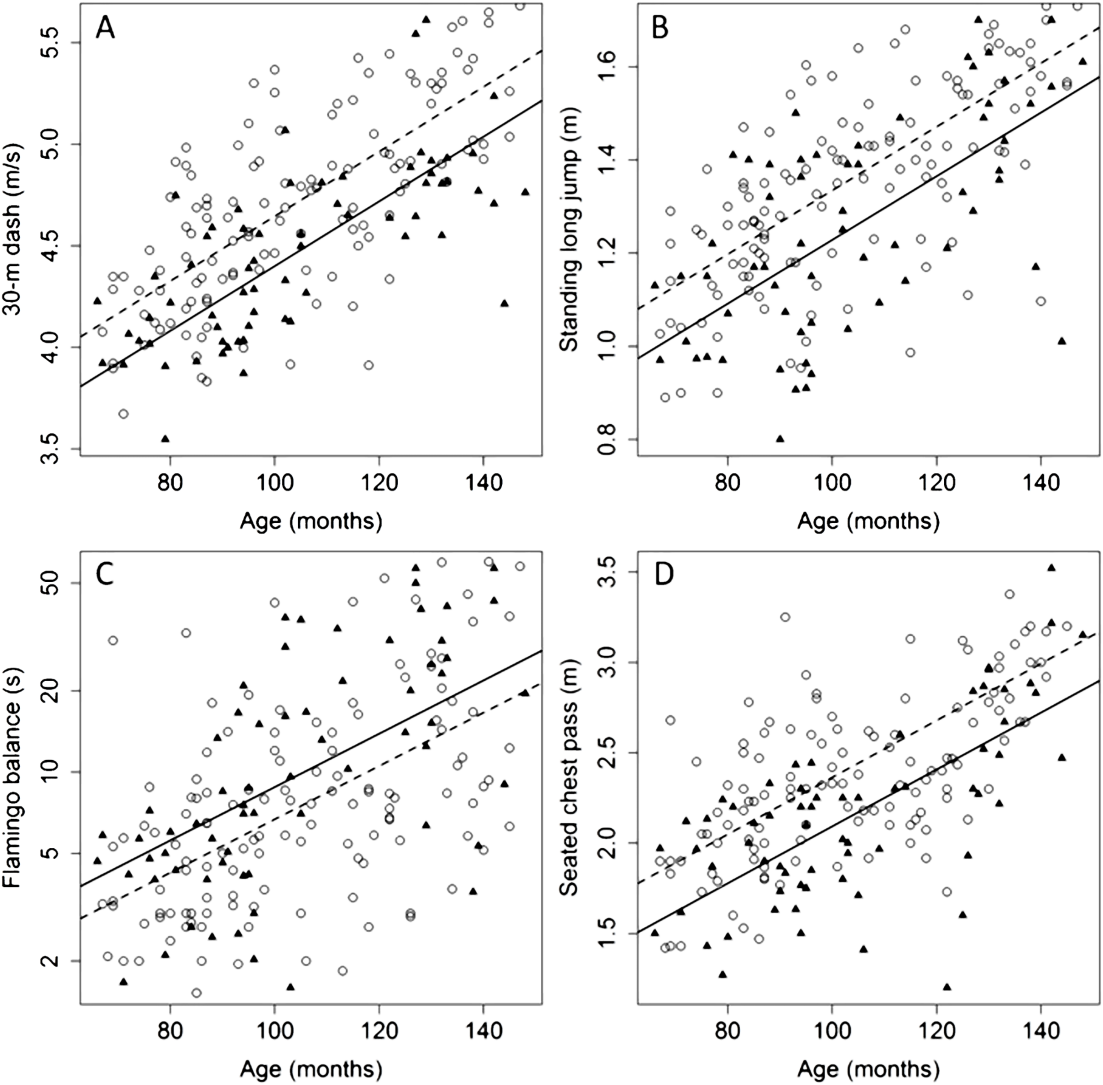
Relationships between age and average performance in four physical fitness tests (A–D) for female and male children aged 6–12. Observed values are plotted using triangles and circles for male and female children, respectively. Solid and dashed lines display the predicted values of the linear regression models in the female and male group, respectively.

The pairwise correlation matrices between putative explanatory variables and performance in individual fitness tests for male and female children stratified by age (≤7 (*n* = 60), 7 < age ≤ 9 (*n* = 64) and >9 (*n* = 69)) are presented in Fig. S1. In the whole study group, age explained 45.2%, 43.6%, 35.6% and 25.6% of the variability for the 30-m dash, seated chest pass, standing long jump, and flamingo balance test, respectively. After removing the effect of age, sex explained 9.5%, 10.7%, 6.3% and 2.0% of the variability for the 30-m dash, seated chest pass, standing long jump, and flamingo balance test, respectively. The relationship between age and average performance in the four fitness tests is shown in Fig. 1 for male and female subjects. To check for possible age-dependent sex-related differences in performance we carried out separate analysis for each of the four tests by first grouping children according to an age cutoff (10 years) and then exploring the interaction effect of age and sex in the two groups. Such an effect was not significant for all tests (*P* value ranging 0.28–0.71).

**Figure 2:**
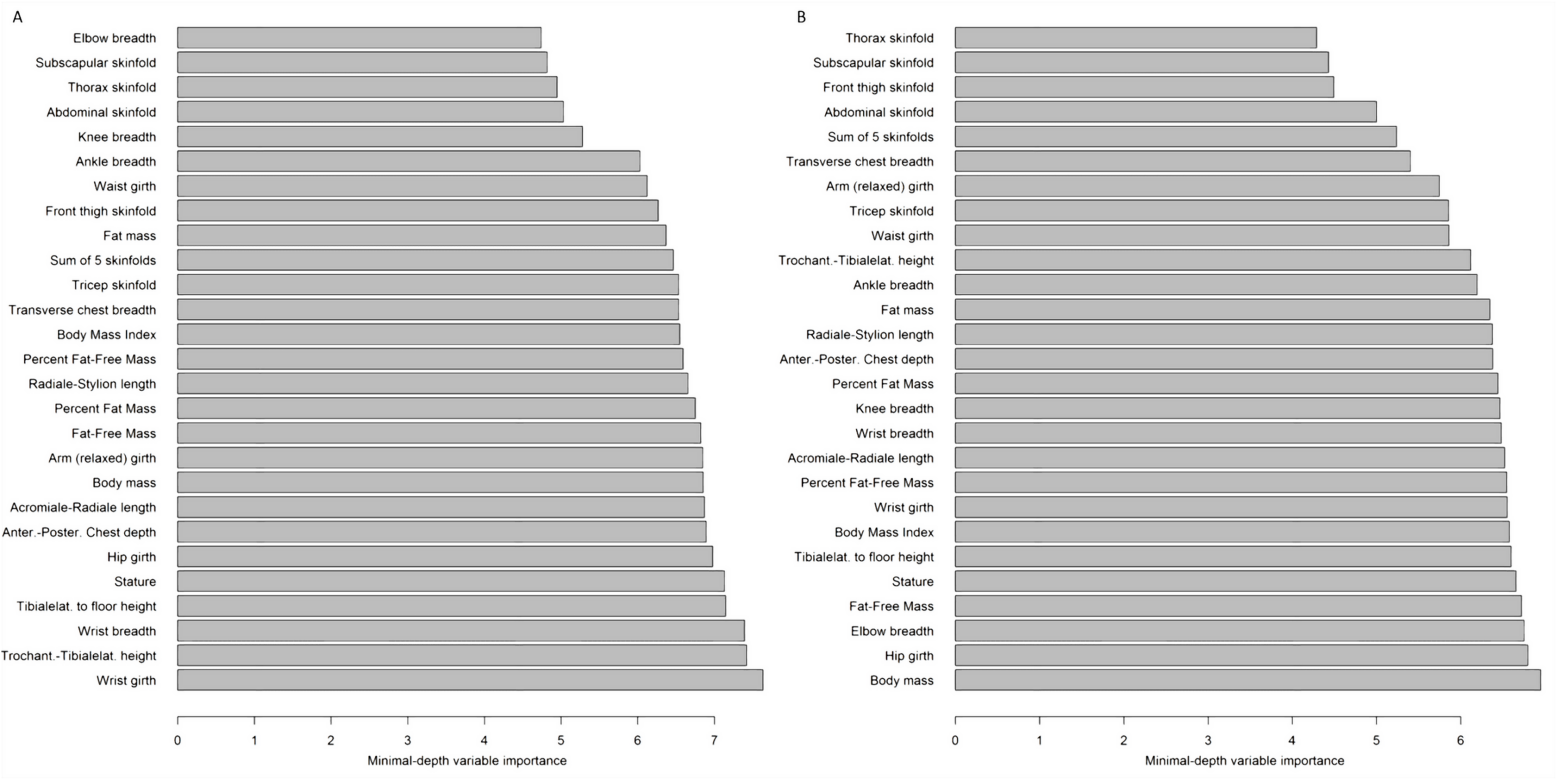
Representative results of Random Forests predictive variable selection. Predictors of average performance in the 30-m dash (A) and in standing long jump (B) tests after the removal of age and sex effects; variables are ranked by minimal depth variable importance according to Random Forests analysis. Lower values indicate higher predictive power.

After removal of the main effect of age and sex, the predictive power of anthropometric variables was estimated using RFs (Fig. 2). A set of predictive variables including skeletal dimensions and a few skinfolds revealed limited predictive power for the 30-m dash and standing long jump test (${R^2} = 3.3{\rm \% }$ and ${R^2} = 4.4{\rm \% }$, respectively; Table 3A). For the seated chest pass and the flamingo balance tests the selected variables were all uninformative (${R^2} = - 5.7{\rm \% }$ and $- 3.4{\rm \% }$, respectively).

**Table 3:** (A) Percent of variance in physical fitness tests explained (out-of-sample) by Random Forests-selected anthropometric variables after removal of the effect of age and sex. (B) Predictive equations for performance of children aged 6–12 in four physical fitness tests.

(A)				
	Physical fitness test			
	30 m-dash	Standing broad jump	Flamingo balance	Seated chest pass
Variable	Elbow breadth Subscapular skinfold Thorax skinfold	Thorax skinfold Subscapular skinfold Sum of five skinfolds Abdominal skinfold	Knee breadth Ankle breadth	Elbow breadth Transverse chest breadth Arm circumference (relaxed) Front thigh skinfold
Explained variance (%)	3.3	4.4	−3.4	−5.7

(B)			
Fitness test	Equation	Adjusted *R*^2^	RMSE
30-m dash (m/s)	2.808 + 0.016 × (age in months) + 0.245 × (sex: 0 = F/1 = M)	0.55	0.33 m/s
Standing long jump (m)	0.5449 + 0.0068 × (age in months) + 0.1061 × (sex: 0 = F/1 = M)	0.47	0.17 m
Flamingo balance (s)	log (flamingo-balance) = −0.095 + 0.023 × (age in months) − 0.275 × (sex: 0 = F/1 = M)	0.32	0.75 s
Seated chest pass (m)	0.519 + 0.016 × (age in months) + 0.271 × (sex: 0 = F/1 = M)	0.45	0.41 m

The individual predictive ability (independent of age and sex) of stature, body mass, and BMI was negative (${R^2}$ ranging from −14.7% to −1.5%). Similar results as above were obtained when the best performance was considered as the outcome in analysis (${R^2}$ ranging from −18.4% to −6.0%).

Predictive equations developed using age and sex are presented in Table 3B. The adjusted coefficient of determination ranged from 0.32 to 0.55. Addition of anthropometric variables to the model did not improve the coefficient of determination for the flamingo balance and seated chest pass test. For the 30-m dash and the standing long jump test, anthropometric variables improved the coefficient of determination by 0.06 and 0.11, respectively, the resulting equation being as follows:

30-m dash (m/s) = 2.567 + 0.016 × (age in months) + 0.178 × (sex: 0 = F/1 = M) − 0.026 × (subscapular skinfold) + 0.122 × (elbow width) − 0.018 × (thorax skinfold), adjusted *R*^2^ = 0.61, root mean square error = 0.31 m/s;

Standing long jump (m) = 0.6406 + 0.0079 × (age in months) + 0.0718 × (sex: 0 = F/1 = M) − 0.0095 × (thorax skinfold) − 0.0140 × (subscapular skinfold), adjusted *R*^2^ = 0.58, root mean square error = 0.15. *P* value was < 0.001 for all models.

## Discussion

Results of this study, carried out in a convenience sample of nonobese children aged 6–12, demonstrate the following points:

1. Chronological age explains the largest proportion of variance in the four fitness tests.
2. Sex shows limited predictive ability independently of age.
3. After accounting for age and sex, anthropometry and body composition show scarce or no ability to explain performance.

This work expands on previous research investigating determinants of physical fitness in childhood (Pissanos, Moore & Reeve, 1983; Butterfield, Lehnhard & Coladarci, 2002; Burns et al., 2015) by using larger number of participants and/or age range as well as a sophisticated statistical analysis preventing collinearity issues with anthropometric predictor variables. Moreover, this is the first study of its kind in the pediatric Italian population. One could be concerned with the unbalance of males and female children in the sample (about 2:1). While such unbalance does not violate any assumption of linear regression analysis, results obtained in a subsample made up of all female children and an equal number of randomly selected male children showed results superimposable with that obtained in the whole sample. Accordingly, we are confident that findings were not distorted by the unequal number of males and females in the sample.

Chronological age affects motor performance by improving the integration of the central nervous system and the skeletal-muscle system for an intended motor task (Haywood & Getchell, 2001) and can modulate muscular strength in youth (Langendorfer & Roberton, 2002); moreover, an effect of age on children motor performance was shown (Pissanos, Moore & Reeve, 1983; Krombholz, 1997; Butterfield, Lehnhard & Coladarci, 2002; Burns et al., 2015). The current results confirm and expand on previous finding (De Miguel-Etayo et al., 2014; Golle et al., 2015; Zaqout et al., 2016) by showing an overwhelming ability of chronological age to determine performance in fitness tests when considered together with sex, body composition and anthropometry. Accordingly, our findings strongly support the practice of using chronological age as a key criterion when implementing physical activity protocols as well as sports participation in children of both sexes aged 6–12. The effect of age was especially relevant when running speed and upper body muscular power are concerned. The effect of chronological age on changes of running speed during growth has not been extensively studied. However, earlier investigation showed that age positively affects running speed (Branta, Haubenstricker & Seefeldt, 1984; Papaiakovou et al., 2009). Here we show that chronological age is able to predict 45.2% of variance in speed of children aged 6–12 and is therefore a key determinant of this fitness component. The role of age in improving running speed is possibly associated with increasing stride length and frequency, and neuromuscular coordination. The seated chest pass test has been found to be a valid and reliable measure of upper body strength in kindergarten children (Davis et al., 2008) as well as a suitable strength-training tool for children about 11 years of age (Alves et al., 2016). This test uses a “general movement common to many sport skills” (Stockbrugger & Haennel, 2001). A main characteristic of this test is that the seated position minimizes the power commonly generated from the trunk and lower extremities in order to strictly explore upper body muscular power. However, the effect of age on performance in this test has been not investigated in children younger than 10 years of age. Here we show that upper body muscular power is strongly (43.6%) dependent on chronological age, which implies that working loads on the upper body should be carefully tuned in children aged 6–12 to match their actual physical potential. Chronological age explained a substantial amount of variance (36.6%) in the standing long jump scores in agreement with recent data showing an increase in standing long jump scores from 6 to 9 years of age in European children (De Miguel-Etayo et al., 2014). However, chronological age was less efficient in predicting lower body muscular power than running speed and upper body muscular power (Table 3) independently of sex, body composition and anthropometry. This suggests that other variables not investigated in this study such as muscle fiber recruitment or muscular coordination are relevant to performance in this test requiring high degree of neuromechanical coordination. Balance is a key ability underpinning many motor skills (e.g., standing and running) and is therefore worth assessing in children. Unfortunately, measurement of balance is not straightforward in children; sophisticated devices or laboratory measurements can be required, and even standardized tests have shown to have a large amount of variability (Deitz, Kartin & Kopp, 2007). The flamingo balance test is a popular field test to assess static balance because it is easy to administer in several settings. In our sample, the distribution of scores from the flamingo test was skewed with about 65% of the scores being <10 s. Accordingly, the flamingo balance test revealed limited potential to accurately reflect all of the ability levels of 6–12 children’s balance explaining 25.6% of variance in the sample and results should be considered with some caution. However, the current finding is supported by data showing an age-related increase in balance ability in children (Humphriss et al., 2011).

Overall, given the positive association of chronological age and performance in all the four physical fitness tests used in this work, scores in those tests may be considered an index of healthy growth.

In our sample, sex explained about 10% or less of variance in fitness tests for speed and muscular power. Our findings confirm and expand on previous work in Grade 1–3 students (Pissanos, Moore & Reeve, 1983) showing that sex is predictive of flexibility and cardiovascular function explaining about 10% of variance, but not muscular power and speed. Consistently, more recent work (De Miguel-Etayo et al., 2014; Golle et al., 2015; Zaqout et al., 2016) showed variable effect of sex on children’s physical fitness. The predictive ability of sex for static balance was quite limited (2%). However, a trend for girls performing better in the flamingo balance test was found in our sample (*P* = 0.089) in accordance with finding of others (Institute of Medicine (IOM), 2012; De Miguel-Etayo et al., 2014). Overall, the limited role of sex in performance suggests that boys and girls in late childhood may participate equally in motor activities. This suggestion is supported by the finding that sex had similar effect on performance in children aged <10 years and ≥10 years.

It has been shown that body dimensions have some relationship with fundamental motor skills in early infancy (Butcher & Eaton, 1989). However, correlations between somatic dimensions and performance in motor tasks during childhood are generally low (<0.50) (Malina, Bouchard & Bar-Or, 2004) suggesting that body dimensions per se are not a major determinant of performance. Our findings support this view by showing that, after accounting for the effect of age and sex, anthropometry retains little or absent predictive ability in performance-related physical fitness tests exploring different aspects of physical fitness. Accordingly, anthropometry should not be a major criterion when identifying physically fit children for sports selection or assigning physical activity tasks, at least in nonobese children. Beyond body dimensions, growth is characterized by rapid changes in body mass and composition. In this work examining a sample of nonobese children we found that neither body mass or composition (percentage of fat or fat-free mass) are significant determinants of physical fitness after adjusting for chronological age and sex. This is supported by the finding of similar performance in fitness tests between normal weight and the limited proportion of overweight children (about 10%) in the sample. An association between obesity or percentage of body fat was found in previous works (Ara et al., 2010; Ceschia et al., 2016; Szmodis et al., 2019) including a substantial proportion of overweight/obese children in the sample. While investigation of such an association was outside the aim of the current work, the lack of significant differences in physical fitness tests performance between normal weight and overweight children may be explained by the low proportion of the latter in the study sample.

We presented herein a set of cross-validated predictive equations (Table 3B) using easy-to-measure variables (just sex and chronological age) as the predictors All the adjusted coefficients of determination (effect size) were large (i.e., >0.26; Cohen, 1992), indicating that equations are able to capture a substantial part of the overall outcome variability. Further, the SEEs are limited, showing that estimates are of practical use. Accordingly, estimates yielded by the proposed equations can serve for evaluation of fitness tests scores in children aged 6–12; they are therefore of use in different physical activity settings for example, school and after-school activities or leisure and sports activities to assess the level of fitness in order to promote a healthy active lifestyle for children or group them for team sport activity and potentially highlight talent even at a young age. Further, these equations can be used to identify imbalances between the four tested aspects of physical fitness (e.g., obvious individual difference from the estimate in one of the four fitness tests) and implement modifications in children’s physical activity accordingly. Given the outstanding changes children undergo during growth, these equations should be used with much caution outside the age range 6–12.

### Limitations

This work has some limitations that should be acknowledged. First, this is a cross sectional study, thereby lacking potential for identification of cause-effect relationships between the independent and dependent variables. Accordingly, a longitudinal study is underway to better explore causal relationships between age, sex and anthropometry and body composition in physical fitness tests performance. Second, we did not control for the effect of maturation on fitness tests performance. Actually, individuals of the same chronological age can differ markedly with respect to biological maturity (Baxter-Jones, Eisenmann & Sherar, 2005), which in turn, could affect test performance. However, in our sample of children aged 6–12 the percentage of individuals showing body mass, stature, and BMI values above the 75th and 95th percentile for the reference population was quite limited, consistent with a very limited impact of maturation bias. Third, we could not adjust performance data by habitual physical activity. Further, we are not sure that unmeasured confounders for example, dietary intake or genetic factors do not have any influence in our findings. Fourth, motor competence of participant children was not objectively assessed as well as other physical fitness components such as endurance or flexibility. Fifth, we did not assess the socioeconomic status of children which may affect for example, motor coordination and gross motor development.

## Conclusions

In conclusion, this work showed that chronological age and, to a lesser extent, sex are main factors for physical fitness of nonobese children aged 6–12, anthropometry and body composition playing a limited role. The predictive equations presented herein can be useful for the evaluation of several components of physical fitness (speed, musculoskeletal power and balance) in children across a wide range of age.

## Supplemental Information

10.7717/peerj.8657/supp-1 Supplemental Information 1 Raw data.

10.7717/peerj.8657/supp-2 Supplemental Information 2 Name and order number of the variables analyzed in this work. Order number is used in the associated correlograms. In correlograms, the scale on the right indicates the strength of correlation (+1/−1)

## Additional Information and Declarations

### Data Availability

The following information was supplied regarding data availability: The raw measurements are available in the Supplemental Files.
