# Supplementary material for: The role of age, sex, anthropometry, and body composition as determinants of physical fitness in nonobese children aged 6–12
Source: PeerJ. 2020 Mar 17;8:e8657. doi: 10.7717/peerj.8657 (PMC7083165; doi:10.7717/peerj.8657)
Supplement: Supplemental Information 2 — Order number is used in the associated correlograms. In correlograms, the scale on the right indicates the strength of correlation (+1/−1) [file peerj-08-8657-s002.pdf]

Supplementary Fig.1

| Variable (unit) | Order number |
|---|---|
| **Demographic** | |
| Age (mo) | 1 |
| Body mass (kg) | 2 |
| Stature (cm) | 3 |
| Body mass index (kg/m$^2$) | 4 |
| **Skinfold** | |
| Triceps (mm) | 5 |
| Subscapular (mm) | 6 |
| Thorax (mm) | 7 |
| Abdominal (mm) | 8 |
| Front thigh (mm) | 9 |
| Sum of five skinfolds (mm) | 10 |
| **Circumference** | |
| Arm (relaxed) (cm) | 11 |
| Wrist (cm) | 12 |
| Waist (cm) | 13 |
| Hip (cm) | 14 |
| **Lengths** | |
| Acromiale-Radiale (cm) | 15 |
| Radiale-Stylion (cm) | 16 |
| **Height** (cm) | |
| Trochanterion-Tibialelaterale (cm) | 17 |
| Tibialelaterale to floor (cm) | 18 |
| **Widths and Breadth** | |
| Transverse chest breadth (cm) | 19 |
| Anterior-Posterior chest depth (cm) | 20 |
| Elbow breadth (cm) | 21 |
| Wrist breadth (cm) | 22 |
| Knee breadth (cm) | 23 |
| Ankle breadth (cm) | 24 |
| **Body composition** | |
| Fat mass (kg) | 25 |
| Percent fat mass (%) | 26 |
| Fat-free mass (kg) | 27 |
| Percent fat-free mass (%) | 28 |
| **Fitness test** | |
| Standing broad jump (m) | 29 |
| 30-m Dash (s) | 30 |
| Flamingo balance (s) | 31 |
| Seated chest pass (m) | 32 |

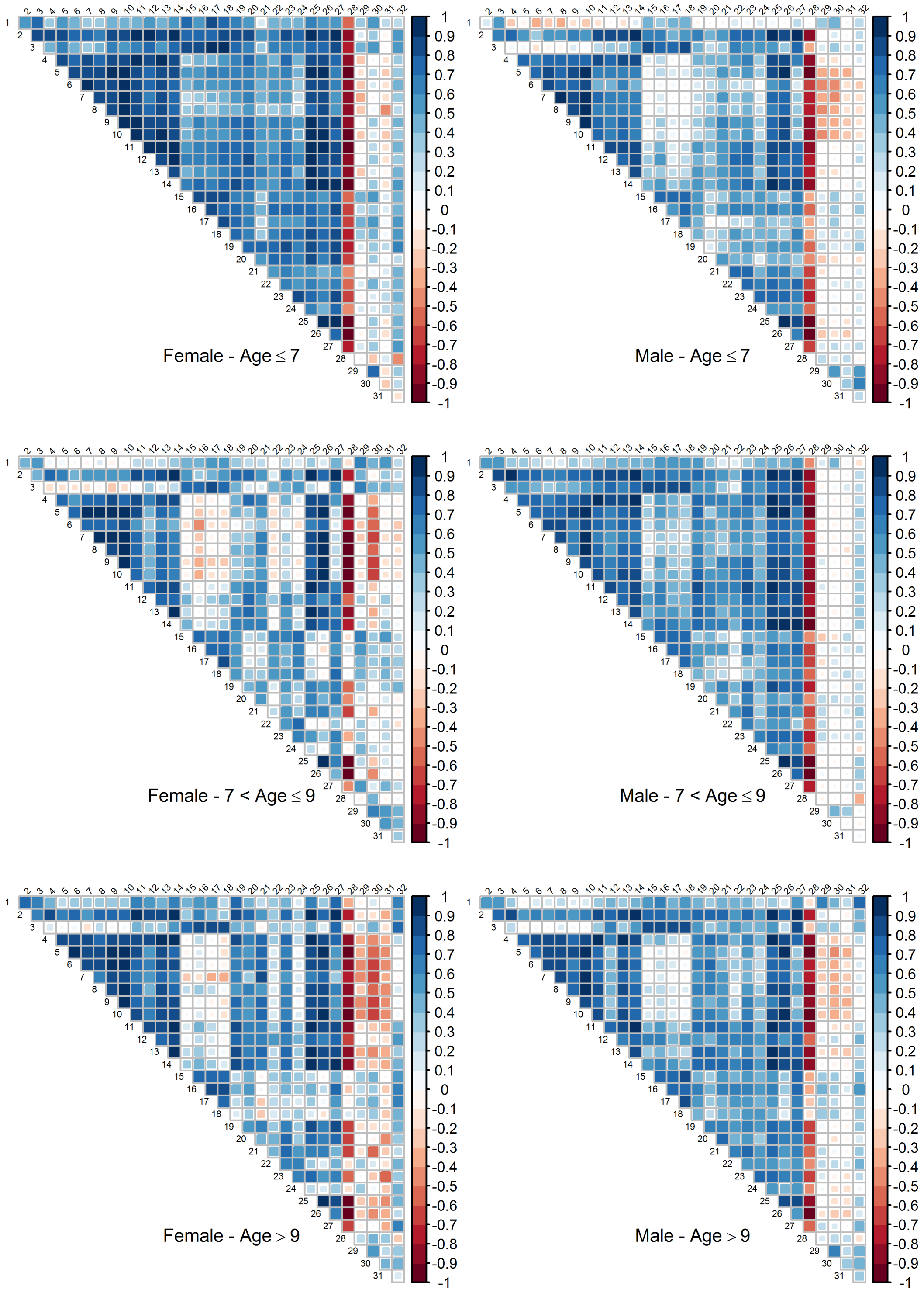
Female - Age ≤ 7
Male - Age ≤ 7
Female - 7 < Age ≤ 9
Male - 7 < Age ≤ 9
Female - Age > 9
Male - Age > 9
